# The Japanese Version of the Functional Listening Index—Paediatric (FLI-P(J)): Translation, Normative Data, and Clinical Feasibility

**DOI:** 10.3390/children13081010

**Published:** 2026-07-30

**Authors:** Jason Hollowell, Tessei Kobayashi, Shigeto Furukawa, Aleisha Davis

**Affiliations:** 1 School of Liberal Arts and Sciences, Musashi University, Tokyo 176-8534, Japan; 2 Speech Language Hearing Center, Shizuoka General Hospital, Shizuoka City 420-0881, Japan; 3 NTT Communication Science Laboratories, Kyoto 619-0237, Japan; 4 Shizuoka Graduate University of Public Health, Shizuoka City 420-0881, Japan; 5 The Shepherd Centre, Sydney, NSW 2042, Australia

**Keywords:** FLI-P, functional listening, hearing impairment, normative data, Japanese children, caregiver report, early childhood, cross-cultural adaptation

## Abstract

**Highlights:**

**What are the main findings?**

• The first Japanese normative percentile curves for the FLI-P(J) were generated from 2512 caregiver reports of typically hearing children aged 2 to 73 months.
• Japanese median and upper-percentile trajectories closely matched existing English-language norms, while the majority of children with reported hearing or language concerns scored at or below the 5th percentile.

**What are the implication of the main findings?**

• The FLI-P(J) provides a culturally grounded, age-referenced benchmark for evaluating functional listening in Japanese children who are deaf or hard of hearing, offering a measurement tool that spans from early prelingual childhood through the preschool years.
• The availability of Japanese percentile curves enables standardized interpretation of functional listening scores for clinical decision-making and supports cross-linguistic comparison with existing English-language norms.

**Abstract:**

**Background**: The Functional Listening Index—Paediatric (FLI-P) tracks functional listening development from birth to six years but lacks Japanese-language norms. This study translated the FLI-P into Japanese (FLI-P(J)) to generate normative developmental trajectories for typically hearing children. **Methods**: Following translation, the FLI-P(J) was administered as an online caregiver survey in Japan. After applying sequential exclusion criteria (removing developmental concerns, zero scores, and IQR outliers) to 2976 responses, a normative sample of 2512 typically hearing children (2–73 months) was retained. Four parameter logistic functions were fitted to empirical percentile trajectories (5th–95th). **Results**: FLI-P(J) scores demonstrated rapid growth across the first three years before gradually plateauing. Phase-level acquisition curves confirmed the expected developmental ordering. Most clinically flagged children scored at or below the 5th percentile. **Conclusions**: This study establishes the first Japanese normative reference curves for functional listening. This cross-linguistic alignment, together with preliminary evidence that clinically flagged children scored at the low end of the distribution, supports the potential of the FLI-P(J) as a clinical benchmark for evaluating Japanese children who are deaf or hard of hearing, pending further validation in independently confirmed clinical samples.

## 1. Introduction

The Functional Listening Index—Paediatric (FLI-P^®^) is a 64-item hierarchical checklist designed to track the development of functional listening skills in infants and young children with hearing impairment. The FLI-P focuses on how children detect, attend to, and make sense of speech and environmental sounds in everyday contexts, rather than on isolated auditory thresholds or decontextualized language test items. The FLI-P was developed at The Shepherd Centre in Australia, in collaboration with the HEARing Cooperative Research Centre, and is intended for use from birth through approximately six years of age [1,2].

The FLI-P comprises 64 items organized into six developmental phases: (1) Sound Awareness; (2) Associating Sound with Meaning; (3) Comprehending Simple Spoken Language; (4) Comprehending Language in Different Listening Conditions; (5) Listening Through Discourse and Narratives; and (6) Advanced Open-Set Listening. It was developed from formative auditory scales in the field of paediatric hearing loss and is intended for use with any child developing listening skills. The full instrument, User Guide, and Item Descriptions are available for clinical use and free download via the developers (The Shepherd Centre/HEARing CRC).

In clinical and early intervention settings, the FLI-P has been shown to be a feasible and informative tool for monitoring listening development in young children with hearing impairment. Davis and colleagues examined its use with a large cohort of children enrolled in early intervention and cochlear implant programs and reported that FLI-P scores could be used to monitor change over time and to support decision-making about intervention [3]. Building on this work, Cowan et al. reported a normative dataset based on parent-reported FLI-P scores for 561 typically hearing children aged 0–72 months [4]. Using quantile regression to model FLI-P scores across age, they generated percentile curves (including the 16th, 50th, and 84th percentiles) that provide age-referenced benchmarks for interpreting the listening development of individual children with hearing impairment.

Although the FLI-P was designed to be conceptually language-independent and has begun to be adapted in other linguistic contexts—for example, through translation and psychometric evaluation in Malay—it has not yet been systematically adapted or validated for use with Japanese-speaking caregivers and children [5]. Cultural and linguistic factors may influence how listening behaviors are expressed, interpreted, and reported by caregivers, and developmental expectations derived from English-speaking or other non-Japanese samples cannot simply be assumed to generalize to the Japanese context. Indeed, direct cross-linguistic comparisons have documented systematic differences in early language acquisition between English- and Japanese-speaking children [6,7,8], underscoring the need for language-specific normative data rather than reliance on translated English-language benchmarks. Establishing Japanese data is therefore an important step before using the FLI-P to support early intervention decisions in Japan, particularly as Japan’s publicly funded newborn hearing screening program has recently expanded, leading to earlier identification and intervention for children with congenital hearing loss [9].

The present study addresses this gap by translating and adapting the FLI-P into Japanese and administering it in survey format to caregivers of children with typical hearing across early childhood. Using an online platform, responses from 2976 caregivers of children aged 2 to 73 months were collected. The study had two aims. The first was to document, for each chronological age, the proportion of Japanese-speaking children whose caregivers report that they consistently demonstrate each listening behavior, thereby generating preliminary normative benchmarks for functional listening development in the Japanese context. The second was to evaluate whether the FLI-P(J) could be feasibly administered via an online, self-completed caregiver survey format and whether the resulting data yielded a robust and interpretable trajectory. After data cleaning and exclusion of responses judged invalid or extreme, age-stratified response patterns were analyzed to generate preliminary developmental benchmarks for Japanese speaking children with typical auditory development. These benchmarks can then be compared with the normative trajectories reported by [4] and, in future work, used to interpret FLI-P scores from Japanese-speaking children who are deaf or hard of hearing.

## 2. Materials and Methods

### 2.1. Translation and Adaptation

Following established guidelines for the cross-cultural adaptation of self-report measures [10], the original English version of the FLI-P was translated into Japanese by a team of three in-house professionals with experience in auditory-verbal therapy and early intervention; two were bilingual (Japanese–English) and produced the initial translation, while the third, a native Japanese speaker, reviewed and refined the resulting Japanese text for naturalness, clarity, and contextual accuracy. Following a forward-translation process, feedback was collected from three Japanese clinicians in Shizuoka General Hospital and two volunteer caregivers of children with hearing impairment to ensure clarity, naturalness, and conceptual equivalence of each item. Revisions were made iteratively based on their feedback to improve comprehension and cultural relevance. No formal cognitive testing (e.g., structured think-aloud protocols) was undertaken; however, clinicians and caregivers were directly asked about item comprehension and naturalness during the review. Minor adjustments were made to item wording and examples to reflect common Japanese expressions and environmental contexts, without changing the core construct being measured. Finally, an independent back-translation into English was conducted and reviewed against the original items by a team of early intervention experts at The Shepherd Centre, including one of the original FLI-P developers (A.D.), to verify the accuracy and conceptual fidelity of the Japanese version.

To illustrate the developmental progression assessed, representative items from the canonical English FLI-P are provided for each phase. Phase 1 (Sound Awareness): “Jumps or startles to loud sounds” (1.1) and “Hears all of the ‘Ling 6’ sounds when presented with emphasis” (1.5). Phase 2 (Associating Meaning): “Knows the voices of 2 family members” (2.3) and “Knows what is going to happen next in familiar songs” (2.8). Phase 3 (Simple Language): “Understands a word or phrase without any actions or gestures” (3.2) and “Knows their own name and will look at me when I say it” (3.4). Phase 4 (Different Conditions): “Follows short directions that are unpredictable or silly” (4.1) and “Repeats all of the ‘Ling 6’ sounds accurately” (4.7). Phase 5 (Discourse): “Recognises a familiar person on the phone” (5.1) and “Follows 3 instructions in the same sentence” (5.10). Phase 6 (Advanced): “Remembers 4 things that happened in a story in the right order after reading a book” (6.3) and “Is able to follow a long, complicated instruction that has more than 5 components” (6.6).

### 2.2. Survey Design and Distribution

The translated FLI-P(J) was administered as part of a larger online survey conducted, all in Japanese, via the commercial market research company ASMARQ Co., Ltd. (Tokyo, Japan), which maintains a large panel of family caregivers in Japan. This study was approved by the institutional review board of NTT Communication Science Laboratories (Approval No. R02-011) prior to data collection. All caregivers provided electronic informed consent before accessing the questionnaire, and participation was entirely voluntary. Survey data were stored on encrypted servers with access restricted to the research team, and no identifying personal information was collected. Panel members receive modest financial incentives for completing surveys. Caregivers of children between 2 and 73 months of age were invited to participate in the survey and only one response per child was permitted. The version of the FLI-P(J) used for this survey was a pre-final Japanese version that differs only stylistically from the version currently available from The Shepherd Centre online (e.g., minor wording and formatting adjustments) and does not change the intended meaning or developmental ordering of any item. The complete Japanese translation of the survey instrument (FLI-P(J)) used in this study is available online in the accompanying Mendeley Data repository [https://doi.org/10.17632/jcvw93k8kj.1].

The FLI-P(J) items were embedded within a broader questionnaire that collected detailed information about the child and family context. Demographic items included, for example, the child’s sex, age and date of birth, health-related diagnoses (e.g., hearing or language development concerns), language(s) spoken in the home, the respondent’s relationship to the child (e.g., mother, father), presence and birth order of siblings, total number of household members, preschool or daycare attendance, history of newborn hearing screening, prefecture of residence, annual income of the primary earner, parents’ ages, employment status (full-time, part-time, or none), and highest level of schooling for both mother and father.

Respondents were geographically distributed across all 47 prefectures of Japan, with the largest single-prefecture contributions from Tokyo (10.5%), Aichi (8.4%), Osaka (8.0%), and Kanagawa (6.7%); no prefecture accounted for fewer than nine responses. Reported annual household income spanned the full range surveyed, from under ¥2 million (3.5%) to ¥15 million or more (1.8%), with the largest single bracket (16.2%) falling at ¥5–6 million, indicating a broad cross-section of household income levels rather than a narrow or skewed subset.

Following the FLI-P(J) section, additional items probed the child’s home literacy and activity environment. Caregivers were asked about how often someone in the family reads to the child, at what age shared reading began, the approximate number of picture books and other reading materials in the home, familiarity with and ownership of specific children’s books, recent play themes and songs the child enjoys, participation in structured activities (e.g., parent-child classes, swimming, dance, Kumon, piano, etc.), the child’s expressive vocabulary using a list of provided words, and the frequency of library use. Although these variables provide rich contextual information, the present analysis focuses exclusively on responses to the FLI-P(J) items. Associations with demographic and home-environment measures will be examined in separate reports.

The survey was distributed with the goal of obtaining responses representing each month of age from 2 months to 73 months (6 years, 1 month). Specifically, the target was 20 males and 20 females per month age group (40 per group). Once 20 children of each gender were recorded for a given month bin, the system automatically stopped accepting additional responses for that group. However, in a few cases, slightly more than 20 were accepted due to near-simultaneous submissions, while in others the total number of submissions was lower due to an automatic global survey closing date.

### 2.3. Administration and Scoring of FLI-P(J) Items

Within the survey, the 64 FLI-P(J) items were presented in their standard developmental order. Items are grouped into phase units, progressing from Phase 1 (Sound Awareness) through Phase 6 (Advanced Open Set Listening), reflecting increasing complexity of functional listening and spoken language skills. Each item describes an everyday listening or language-related behavior (e.g., “jumps or startles to loud sounds”), and caregivers were asked to indicate how often their child currently demonstrates that behavior by selecting either “Mostly” or “Rarely.” The full instrument is available for reference via the HearHub platform operated by The Shepherd Centre in Australia [2].

In typical clinical administration of the FLI-P, item presentation is discontinued once a child receives six consecutive lower-frequency responses (e.g., “Rarely”). Because this survey was completed independently by caregivers in an online format, it was not practical to expect respondents to keep track of these ceiling and basal rules and discontinue the questionnaire themselves. For this reason, all caregivers were asked to respond to every FLI-P(J) item, regardless of their child’s performance.

Total FLI-P(J) scores were then derived post hoc using a rule designed to approximate the original stop protocol. Responses were coded dichotomously, with “Mostly” treated as indicating that the behavior was present and “Rarely” as indicating that it was not yet consistently observed. For each child, the FLI-P(J) total score was calculated as the number of “Mostly” responses counted up to the point at which six consecutive “Rarely” responses occurred; any “Mostly” responses after this run of six “Rarely” responses were not included in the total. This procedure yields a single total score per child that is comparable in interpretation to scores obtained under standard FLI-P administration, while accommodating the constraints of self-administered online data collection.

### 2.4. Participant Characteristics and Data Cleaning

A total of 2976 responses were collected across all age groups. Eligibility for the normative analysis was restricted to children without reported hearing, language, or related developmental concerns based on screening questions. Data cleaning was performed in three sequential steps:

1. Screening for caregiver-reported suspected developmental concerns:
  Responses were excluded if the caregiver selected any response other than “None in particular” (Original—特になし) for the question:
  “Please tell us if your child has ever been identified as having any concerns related to sensory functioning or behavior during health checkups or visits to medical institutions.”
  Original—「健診や医療機関などでお子様の感覚機能や行動面などでこれまでに指摘された点があれば教えて下さい。」
  The options, with number of responses removed for each, were:
  1. No concerns reported (特になし)           = All retained
  2. Auditory function/hearing (耳の聞こえ/聴覚機能)  = 9
  3. Visual function (視覚機能)             = 1
  4. Language development (ことばの発達)        = 32
  5. Social functioning (社会性)            = 6
  6. Attention and behavioral regulation (注意/落ち着き)   = 0
  7. Other (その他)                   = 0

This screening removed 48 responses that reported prior concerns related to hearing, vision, speech development, social behavior, attention, or other domains. These classifications were based solely on caregiver reports in response to this screening question; no independent clinical verification or detailed diagnostic information was obtained as part of the survey.

2. Exclusion of implausible total scores:

A total of an additional 263 response sets with a score of 0 were removed. In the FLI-P, a total score of 0 indicates that the caregiver reported “Rarely” for all of the early items, including very basic behaviors such as startling to loud sounds or responding to familiar voices. Given the simplicity of the earliest items, such a pattern was deemed implausible for any child regardless of age, and these responses were interpreted as likely indicating non-serious participation, possibly submitted solely for remuneration.

3. Outlier removal using the Interquartile Range (IQR) method:

Finally, to reduce the impact of extreme under- or over-reporting, the Interquartile Range (IQR) method was used to identify and remove outliers based on total score distributions within six-month age bands. Because the youngest children in the dataset were 2 months old and the oldest were 73 months, twelve bands of equal width: 2–7, 8–13, 14–19, 20–25, 26–31, 32–37, 38–43, 44–49, 50–55, 56–61, 62–67, and 68–73 months (corresponding to the age bands shown in Table 1) were defined. For each age band, the first quartile (Q1), third quartile (Q3), and the IQR (Q3–Q1) for total FLI-P(J) scores were calculated. Observations with scores below Q1 − 1.5 × IQR or above Q3 + 1.5 × IQR were treated as outliers and excluded from further analysis. The IQR-based approach is widely used in applied research because it relies on medians and quartiles rather than means and standard deviations and is therefore robust to skewed or non-normal distributions—conditions that are typical in developmental data where floor and ceiling effects are expected. In addition, recent work has recommended IQR-based trimming as a practical and effective strategy for outlier detection in large-scale empirical datasets, where extreme values can distort model estimates if left unaddressed [11]. The symmetric IQR method was selected because it treats both tails equally, applying the same principled, distribution-based criterion at each end regardless of the direction of response bias (e.g., over-reporting vs. under-reporting), rather than relying on a fixed percentile cutoff at one end only. As detailed below, this approach was compared against an alternative that combined a fixed 5th-percentile cutoff at the low end with an IQR-based fence at the high end; the two methods yielded nearly identical developmental trajectories, and the symmetric IQR heuristic was retained as the primary approach for its methodological consistency and transparency. This step excluded an additional 153 response sets from the analysis.

As a robustness check, an alternative outlier removal method was also explored, in which scores below the 5th percentile were excluded at the low end and the IQR method was applied only at the high end to detect extreme scores. This approach removed 164 responses rather than 153. As described in the Results, the developmental trajectories obtained under the two cleaning strategies were highly similar, indicating that the specific choice of exclusion threshold did not materially affect the observed pattern of age-related change. On this basis, the standard symmetric IQR-based method was adopted for the final analyses reported in this paper, resulting in a final normative sample of N = 2512 caregiver reports.

**Figure 1:**
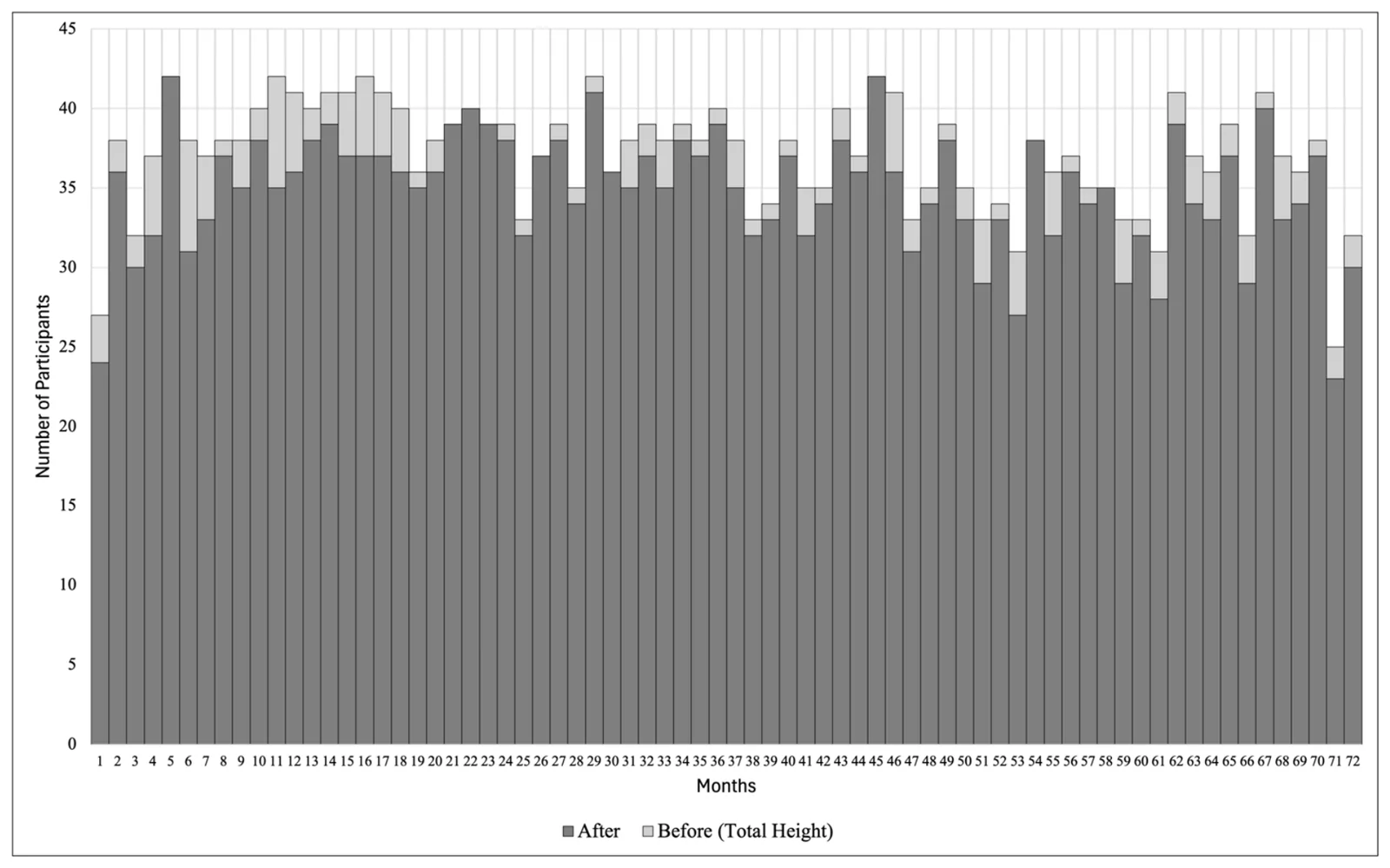
Number of caregiver responses per month of age, before and after data cleaning. The full bar height shows the original number of responses; the dark segment shows responses retained after cleaning; the light segment shows responses removed.

To illustrate how the cleaning steps affected the usable sample at each age, the number of caregiver responses per six-month age band before and after the final IQR-based outlier removal were summarized (Table 1). The first numeric column shows the total number of responses collected in each band; the second shows the number of cases retained for the normative analysis after medical screening, exclusion of zero scores, and removal of outliers using the IQR criterion; and the third reports the corresponding percentage retained. As a complementary view, for each individual month of age, the number of caregiver responses before and after data cleaning, were also plotted (Figure 1). The specific purpose of Figure 1 is to allow verification of sampling fidelity to the target design (20 males and 20 females per month); the monthly resolution confirms that recruitment remained balanced across the age range without significant monthly gaps. This month-by-month display highlights local fluctuations in both the volume of data and the number of excluded cases, while confirming that the retained sample remains substantial at each age and that outlier exclusion did not disproportionately reduce the number of usable cases at any particular age.

### 2.5. Statistical Analysis

Initial data screening, calculation of age-band indicators, and implementation of outlier-removal rules (IQR- and percentile-based thresholds) were conducted in Microsoft Excel. These steps included computing age bins, identifying zero scores, and applying the interquartile range (IQR) fences and alternative 5th-percentile thresholds at the age-band level.

All subsequent descriptive and graphical analyses were carried out using jamovi, an open-source statistical platform built on the R language [12]. Jamovi 2.7 was used to generate descriptive statistics (means, medians, and selected percentiles) for FLI-P(J) total scores by age in months and by age bands, and to produce scatterplots and smoothed developmental trajectories.

To model the age-related trajectories, we evaluated several smoothing approaches, consistent with established methods for constructing age-based percentile reference curves [13]. A four-parameter logistic function was selected for all curve fitting because it ensures methodological consistency with the original English-language study [4] and provides biologically plausible asymptotes at the floor and ceiling of development. We also assessed a locally weighted scatterplot smoothing (LOWESS) approach as a non-parametric alternative; however, the logistic function was retained for the final models because it is more parsimonious and directly comparable to the Cowan et al. norms.

The 5th, 10th, 16th, 50th, 84th, 90th, and 95th percentiles were selected for analysis. The 16th and 84th percentiles correspond approximately to ±1 standard deviation from the mean in a normal distribution, defining the range of typical development. The 5th and 10th percentiles are included as progressively stringent clinical risk indicators often used in developmental screening, and the 90th and 95th percentiles mark the upper extreme. This selection mirrors those reported by Cowan et al. [4], thereby facilitating direct visual comparison between the English-language and Japanese norms.

The primary outcome variable for analysis was the child’s total FLI-P(J) score after data cleaning, with age in months treated as a continuous predictor. To visualize developmental change across early childhood, individual data points were fitted and plotted using the four-parameter logistic curves from the Cowan study to model the trajectory of functional listening development over time [4]. In addition, for each month of age empirical percentiles were calculated (5th, 10th, 16th, 50th, 84th, 90th, 95th) and then fitted using four-parameter logistic functions to the age profiles of these percentiles, yielding a set of logistic percentile curves that illustrate the spread of scores around the central trajectory.

In an additional item-level analysis, acquisition probabilities were calculated for selected items and for phase-level item averages within each six-month age band, defining acquisition as caregiver endorsement of an item as “Mostly”.

## 3. Results

### 3.1. Effects of Screening

After the initial medical screening and removal of implausible zero scores, as referenced above, two alternative outlier-handling approaches were applied and compared. In the first approach, scores below the 5th percentile within each age band were removed at the low end, and IQR-based fences were applied only to identify high-end outliers. In the second approach, IQR-based fences were applied symmetrically at both the low and high ends of the distribution within each age band.

**Figure 2:**
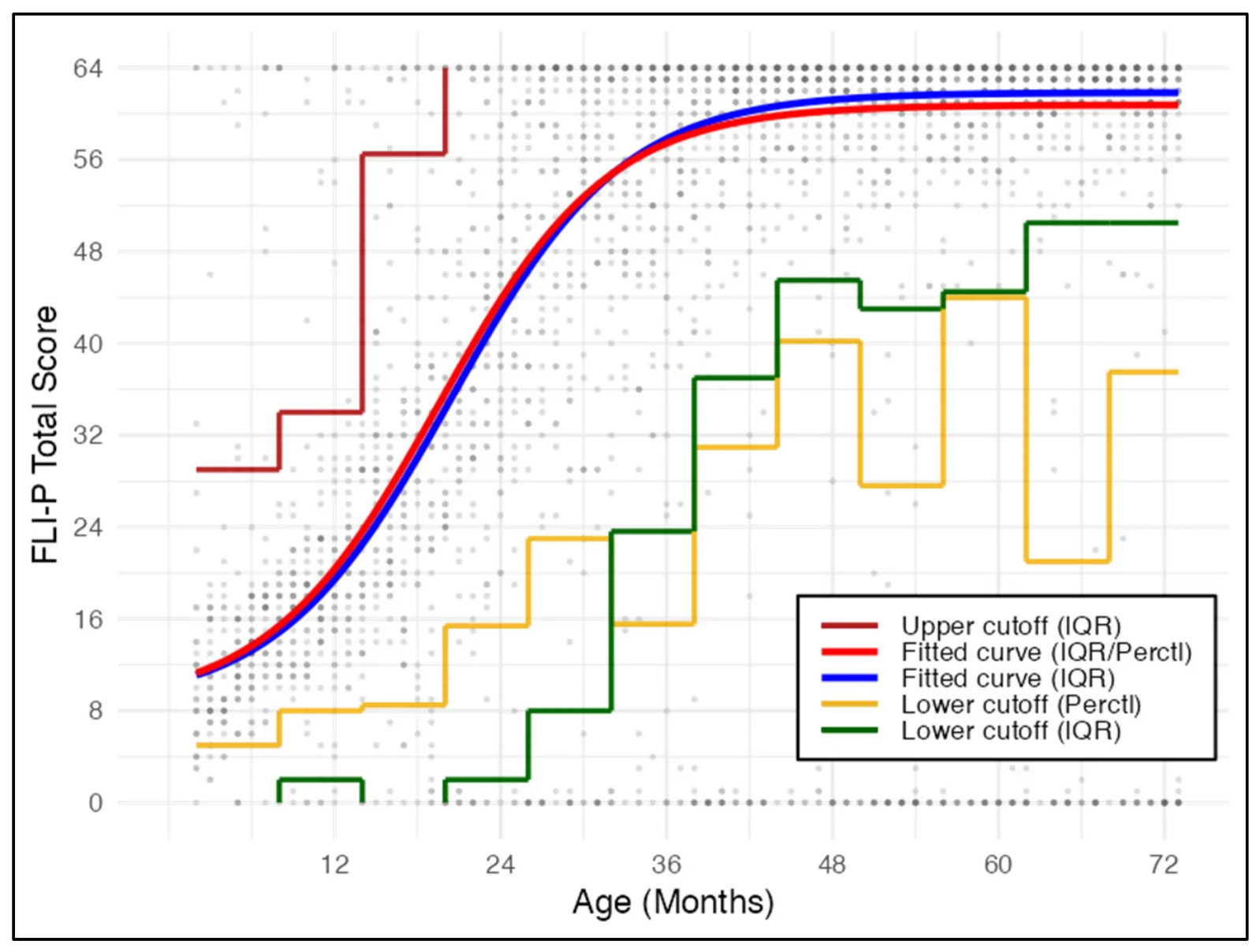
Developmental trajectories of FLI-P(J) total scores under the two outlier-handling strategies described in Section 2.4, with age-band-specific exclusion thresholds overlaid. Points are shaded from light grey to black according to the number of overlapping observations at each score; all observed scores prior to outlier removal are shown; the blue curve shows the symmetric IQR fit, and the red curve shows the 5th-percentile/IQR fit. Yellow, green, and red step lines mark the 5th-percentile, lower IQR, and upper IQR thresholds, respectively.

As summarized in Table 1, both strategies yielded a large cleaned normative dataset (N = 2512) with high retention across age bands from 2 to 73 months. Retention rates (Table 1) varied modestly by age, ranging from 77.87% (68–73 months) to 90.44% (20–25 months). This pattern may partly reflect greater score compression near the upper end of the scale in older children, which narrows the interquartile range and increases the likelihood that individual scores are flagged by the IQR fences; we did not, however, systematically investigate the source of this variation, and retention remained above 75% in every age band. To visualize how these choices affect the developmental trajectory of FLI-P(J) scores, separate four-parameter logistic curves were fitted to the retained cases under each method and plotted over the full set of observed scores (Figure 2). The blue curve represents the symmetric IQR-based method (upper and lower fences), and the red curve represents the alternative method combining a 5th-percentile cutoff at the low end with an upper IQR fence.

Figure 2 also overlays the age-band-specific exclusion thresholds used in the cleaning process. For each six-month band, yellow step lines mark the 5th-percentile cutoffs, dark green lines indicate the lower IQR bounds, and dark red lines indicate the upper IQR bounds, with vertical segments connecting adjacent bands and indicating where thresholds fall off the plotted scale. Together, these elements show how the two outlier-handling rules operate across age and where responses were trimmed from the lower and upper tails of the distribution.

Visual inspection of Figure 2 indicates that the logistic trajectories obtained under the two methods are closely aligned across the age range, with only minor deviations at the youngest and oldest ages. This similarity suggests that the main pattern of age-related change in FLI-P(J) scores is robust to reasonable choices of exclusion threshold. On this basis, the symmetric IQR-based method was adopted as the primary approach for all subsequent analyses reported in this paper. To illustrate the outlier cleaning process, an extraordinarily low score for an older child—for example, a 68–73-month-old performing at a 6–12-month level—would be developmentally implausible for a typically developing child and would be flagged as an outlier by the symmetric IQR criterion.

### 3.2. Growth and Distribution of FLI-P Scores

**Figure 3:**
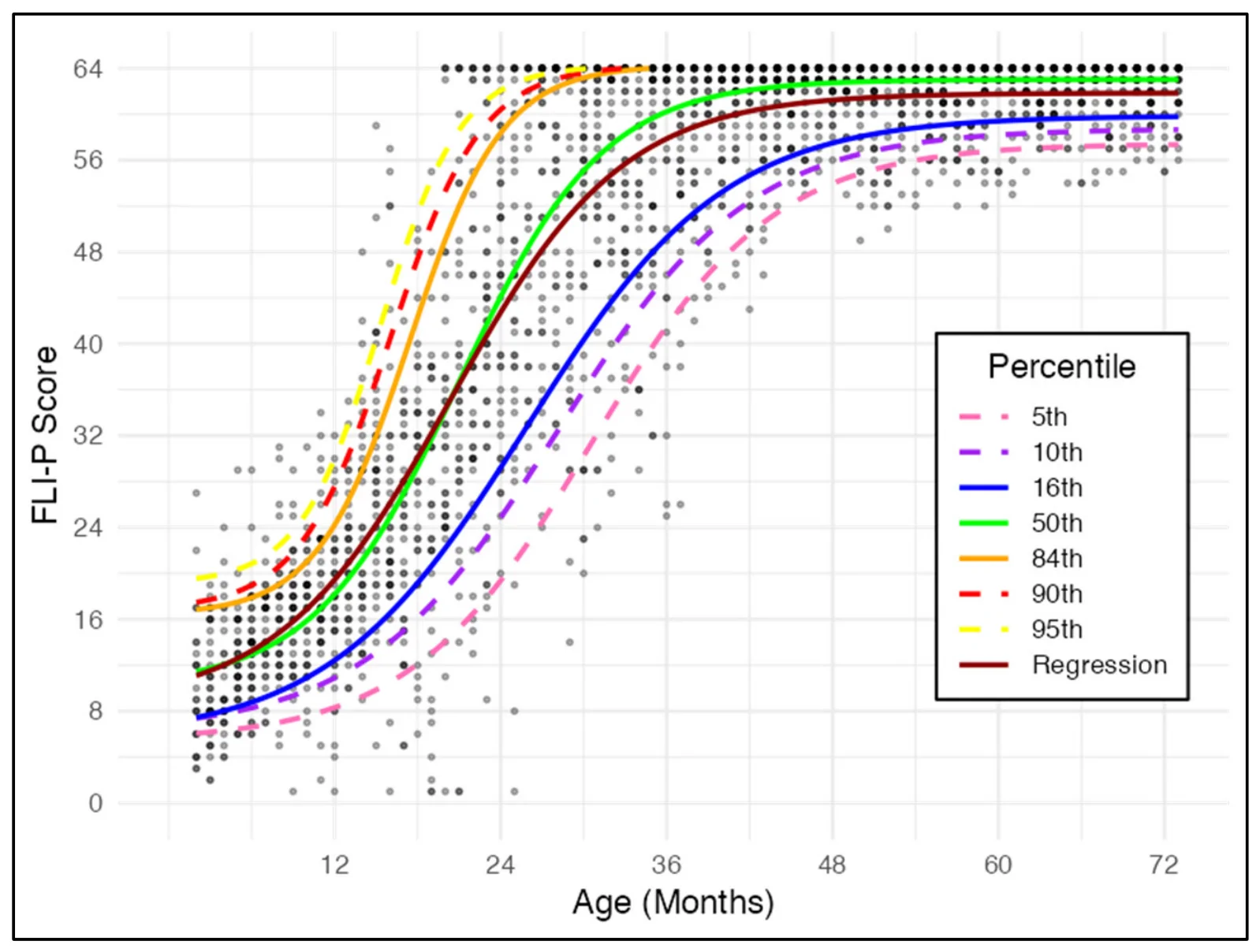
Developmental trajectory of FLI-P(J) total scores for the cleaned normative sample (N = 2512), showing the central four-parameter logistic curve and logistic percentile curves (5th–95th) over individual child scores. Points are shaded from light grey to black according to the number of overlapping observations at each score; only retained scores after outlier removal are shown.

To further characterize the distribution of FLI-P(J) scores across age and to align more closely with the percentile-based presentation used by Cowan et al. [4], age-related percentile curves were then estimated from the cleaned dataset. For each month of age, empirical score distributions were summarized and used to calculate the 5th, 10th, 16th, 50th, 84th, 90th, and 95th percentiles. To obtain smooth age-related trajectories, four-parameter logistic functions were then fitted to the age profiles of each percentile, yielding a set of logistic percentile curves together with a central four-parameter logistic curve for the full cleaned sample (Figure 3).

In Figure 3, individual data points provide a background display of the observed variability, while the overlaid logistic percentile curves illustrate how the distribution of FLI-P(J) scores shifts with increasing age. The 50th percentile (median) closely tracks the central four-parameter logistic curve, indicating that this fitted function provides a reasonable summary of typical listening development across the age range. The 5th–95th percentile band captures the majority of observed scores at each age, with the upper percentile curves approaching the maximum FLI-P(J) score in the later preschool years, between 4 and 5 years of age, and the lower percentiles remaining low but clearly above zero across the age range, consistent with the expectation that even very young children with typical hearing demonstrate at least some functional listening behaviors.

Taken together, the four-parameter logistic curve and the accompanying percentile trajectories provide complementary views of the data: the central curve emphasizes the overall developmental trend in functional listening, while the percentile curves highlight age-related changes in the spread of scores and support comparison with previously published percentile-based reference curves.

**Figure 4:**
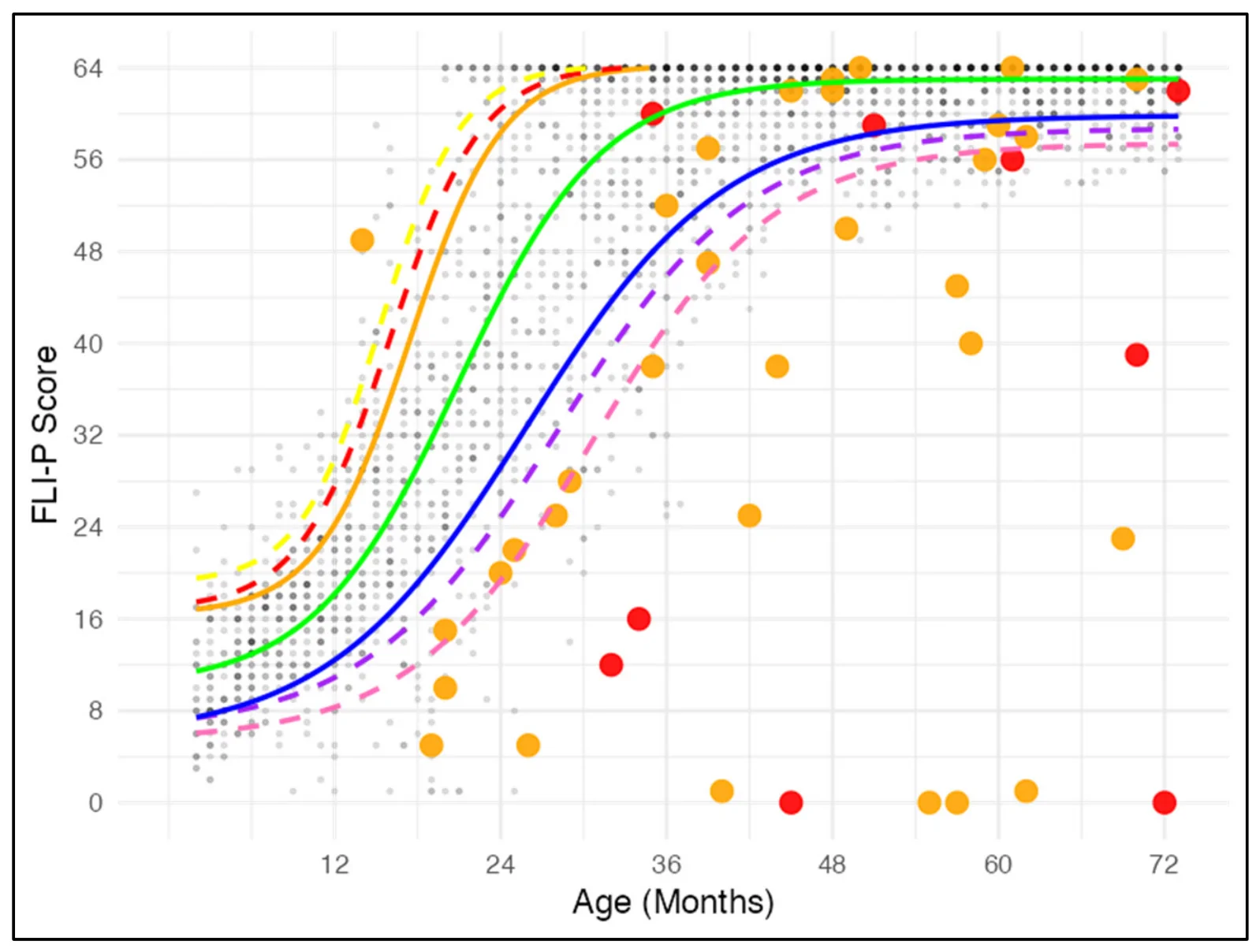
Developmental trajectory of FLI-P(J) total scores with percentile curves (5th–95th) for the typical hearing sample, with overlaid data points representing children flagged on the medical screening question. The background scatter and percentile curves are identical to those in Figure 3; the solid curve lines show the four-parameter logistic fit to the normative sample with the regression line removed in this figure. Red points indicate the nine children whose caregivers reported prior identified concerns about hearing, and orange points indicate the 32 children whose caregivers reported an identified language-development delay. The screening question for these flagged cases asked about “identified” delays or issues during health checkups or medical visits, rather than general parental concern.

To illustrate how the percentile curves may be used to interpret scores for children with known or suspected developmental difficulties, the nine responses from caregivers who reported prior concerns about their child’s auditory function and the 32 responses from caregivers, who reported that their child had been identified as having a delay in language development, were reintroduced into the dataset and overlaid on the normative trajectory as individual points (Figure 4). All of these children had been excluded from the main analyses at the medical-screening stage but are shown here as overlaid markers (red for hearing concerns, orange for language-development delay) to indicate where they fall relative to the four-parameter logistic trajectory and the age-related percentile curves derived from the typical hearing sample.

Visual inspection of Figure 4 shows that 5 of the 9 children whose caregivers reported identified hearing concerns (red) fall clearly below the 5th percentile for their age, one case lies close to the 5th percentile, one on the 16th percentile, and the remaining two cases fall on the 50th percentile (median) curve. Thus, the majority of children with identified auditory difficulties show markedly reduced functional listening scores relative to age-matched peers, although a small subset achieves scores within the typical range.

The same figure also displays the 32 children whose caregivers reported that their child had previously been identified as having a delay in language development (orange). Of these, 21 fall on or below the 5th percentile curve for their age, one lies between the 5th and 10th percentiles, one falls between the 10th and the 16th percentiles, and two lie between the 16th percentile and the central logistic curve. The remaining seven cases fall above the regression line; although only six points are visible in that region, two children share the same score of 62 at 48 months, causing one point to overlap the other. One child of roughly 15 months shows a markedly elevated score, well above the 95th percentile. Thus, most children with a documented history of language delay cluster at the very low end of the normative distribution, while a smaller subset score within the typical range and one child scores markedly above it, underscoring that functional listening outcomes in this group are somewhat heterogeneous rather than uniformly depressed.

Because the present data were collected via an online caregiver survey, we did not obtain detailed audiological information (e.g., degree or configuration of hearing impairment, age at diagnosis, use of hearing technology) or formal diagnostic profiles for language delay. As a result, we are unable to further characterize these outlying cases or examine how severity or intervention history relates to their position on the normative curves. Nevertheless, the fact that the majority of clinically flagged children fall at the low end of the Japanese FLI-P(J) distribution provides preliminary support for the clinical interpretability of the normative curves.

### 3.3. Item Acquisition Patterns Across Developmental Phases

To examine developmental progression across listening skills, acquisition probabilities were calculated at the phase level. For each of the six developmental phases, the probability of item endorsement (“Mostly”) was averaged across all items within that phase and plotted against age. The six developmental phases represented in these analyses correspond to the underlying structure of the FLI-P(J): Phase 1, Sound Awareness; Phase 2, Associating Sound With Meaning; Phase 3, Comprehending Simple Spoken Language; Phase 4, Comprehending Language In Different Listening Conditions; Phase 5, Listening Through Discourse And Narratives; and Phase 6, Advanced Open Set Listening.

**Figure 5:**
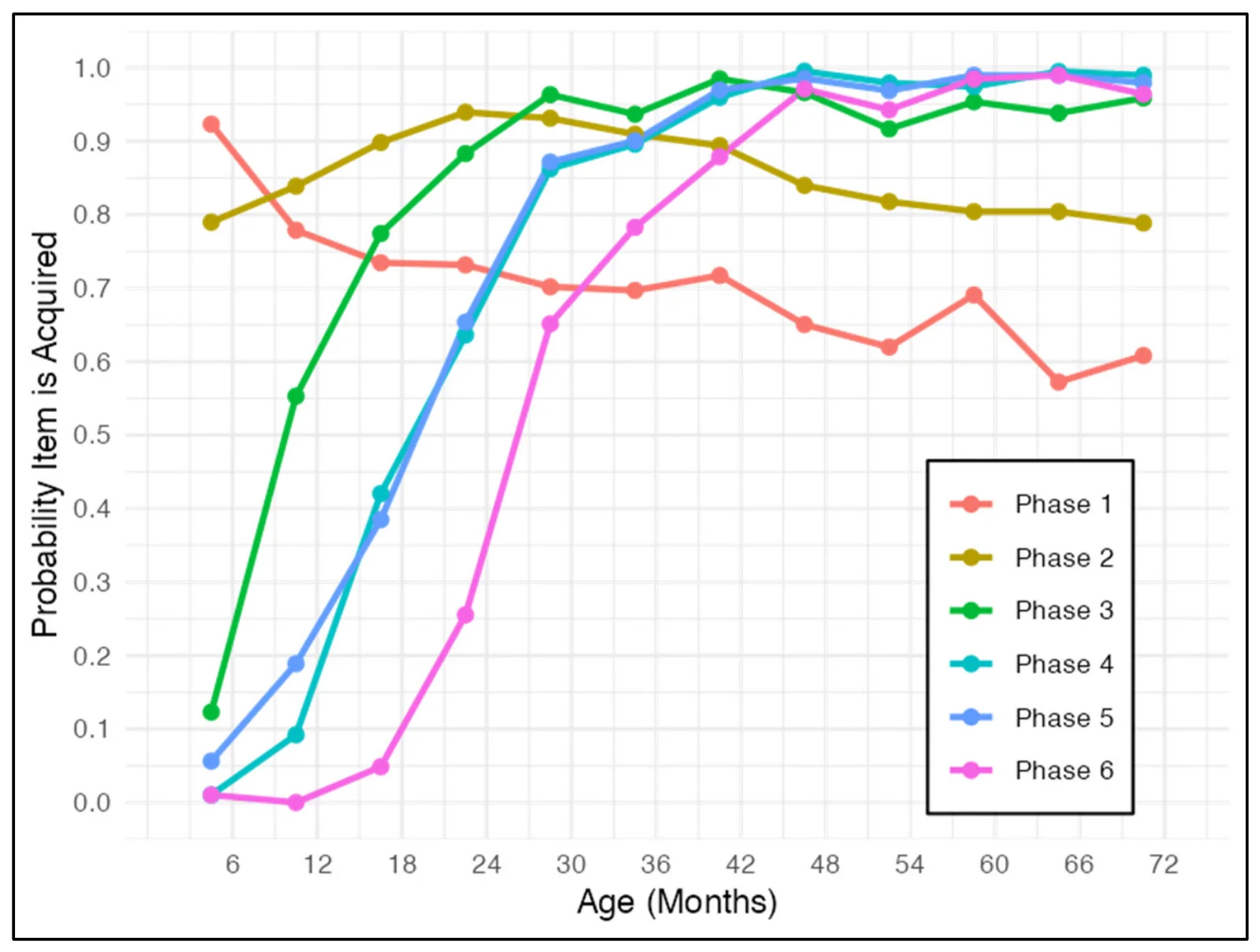
Average item acquisition curves by developmental phase for the FLI-P(J). For each six-month age band, the *y*-axis shows the mean proportion of items within each phase endorsed as “Mostly” by caregivers in the cleaned normative sample.

Figure 5 presents the resulting phase-level acquisition curves. The curves demonstrate a clear and orderly developmental progression, with earlier phases reaching higher levels of acquisition at younger ages and later phases emerging more gradually over time. Phase 1 reaches high acquisition levels earliest, followed in sequence by Phases 2 through 6.

This pattern is consistent with the theoretical structure of the FLI-P framework, in which listening development progresses from basic sound awareness to increasingly complex language comprehension and discourse-level processing. The phase-level curves provide additional support for the developmental validity of the FLI-P(J), indicating that the Japanese version preserves the expected ordering of functional listening skills across phases.

Taken together, these phase-level acquisition patterns complement the total-score analyses by showing that the developmental progression captured by the FLI-P(J) is evident not only in overall scores but also in the age-related emergence of behaviors across the instrument’s underlying phase structure. In Figure 5, the slight decline in Phase 1 and 2 acquisition probability at older ages is interpreted as a reporting artifact rather than developmental regression. Items like “startles to loud sounds” are highly salient in infants but may be less observable or simply ignored by caregivers of older children who have mastered complex conversation. This salience bias does not affect later phases, which measure skills that remain active throughout the preschool years.

### 3.4. Comparison with Existing English-Language Norms

**Figure 6:**
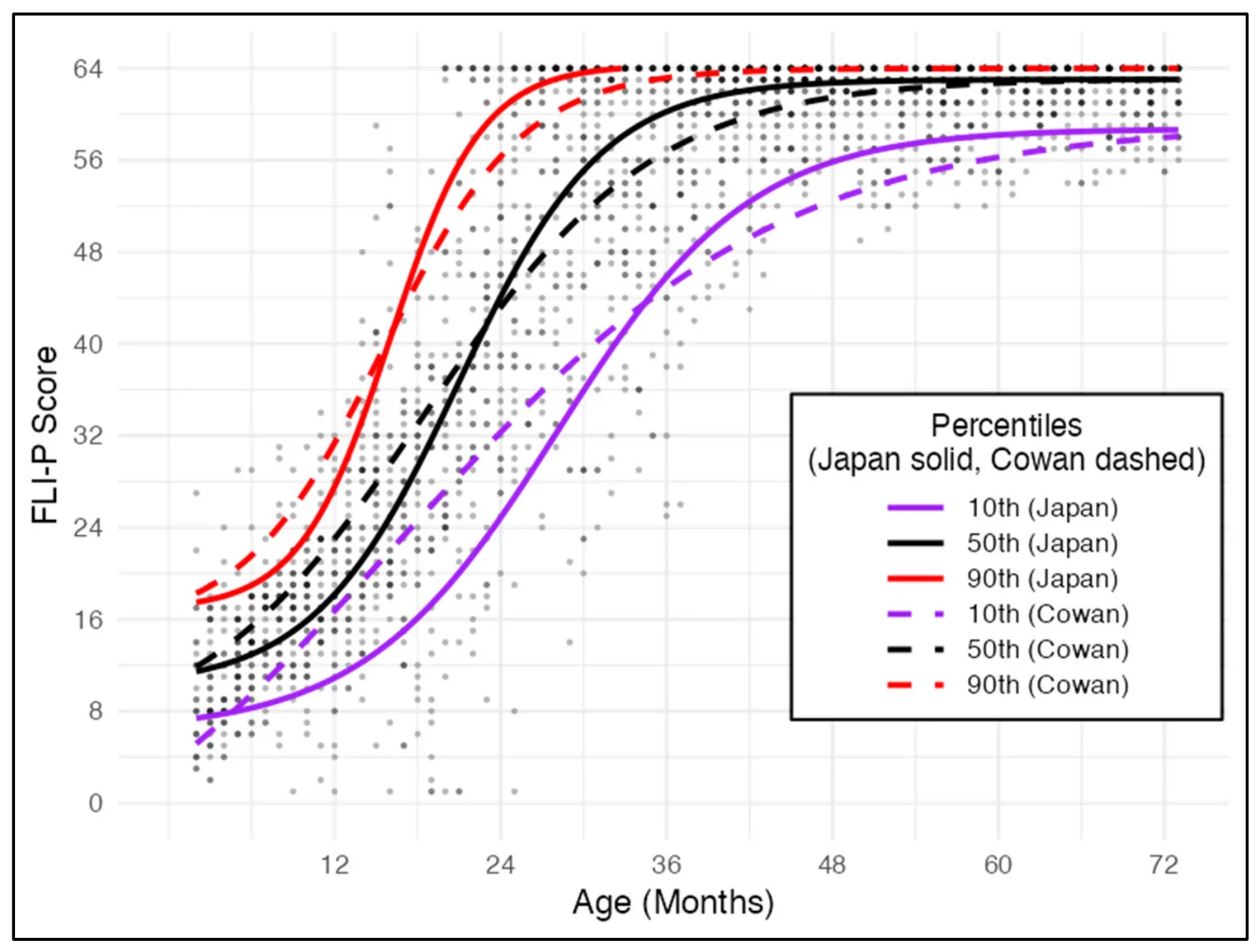
Developmental trajectory of FLI-P(J) total scores showing four-parameter logistic 10th, 50th, and 90th percentile curves for the Japanese normative sample (solid lines) overlaid with the corresponding logistic growth functions reported by Cowan et al. [4] for an English-speaking sample (dashed lines). Points are shaded from light grey to black according to the number of overlapping observations at each score; only retained scores after outlier removal are shown.

To situate the Japanese normative data in relation to previously published benchmarks, four-parameter logistic growth functions were estimated for the 10th, 50th, and 90th percentile trajectories in the present sample and overlaid on the corresponding functions reported by Cowan et al. [4] for typically hearing children in an Australian English-speaking context (Figure 6). In this figure, solid lines represent the Japanese percentile curves derived from the FLI-P(J) dataset, and dashed lines represent the Cowan et al. logistic curves.

Visual inspection indicates that the Japanese 50th and 90th percentile trajectories follow the Cowan functions very closely in both shape and asymptotic level, with only small differences in the timing of the rise and plateau. No formal statistical test of curve divergence was performed; the comparison is based on visual inspection of the overlaid logistic functions. The 10th percentile curves show somewhat greater divergence at certain ages but still exhibit a broadly similar developmental pattern. This pattern of results suggests that the developmental profile of functional listening captured by the FLI-P is highly similar across Japanese- and English-speaking populations at the median and upper range of performance, with broadly comparable trends at the lower end. Together, these findings support both the cross-linguistic applicability of the instrument between English and Japanese and the potential utility of the Japanese norms as a reference for clinical use, pending further validation in independently confirmed clinical samples.

## 4. Discussion

The present study translated, adapted, and systematically evaluated the Functional Listening Index—Paediatric for use with Japanese-speaking caregivers, generating preliminary normative data for the Japanese version (FLI-P(J)). Using caregiver reports from a large online panel and applying a structured, multi-step cleaning procedure, we obtained a final normative sample of 2512 children aged 2–73 months. Four-parameter logistic curves fitted to the age-related percentile trajectories showed the expected developmental pattern: a rapid rise in total FLI-P(J) scores over the first three years of life, followed by a gradual plateau approaching the maximum score in the preschool years. These findings indicate that the Japanese adaptation behaves in a manner consistent with developmental expectations and provides age-referenced benchmarks for functional listening in children with typical hearing.

### 4.1. Establishing a Japanese Normative Baseline

By modelling both a central trajectory and percentile bands (5th–95th) across age, this study provides the first normative reference curves for the development of listening skills in children raised in Japanese-speaking environments using the FLI-P(J). The overall shape of the Japanese developmental trajectory closely parallels the English-language normative curves reported by Cowan et al. [4], with steep early gains followed by a plateau between ages 30 to 48 months. This convergence supports the underlying developmental logic of the FLI-P across languages: as children age, they are expected to show increasingly consistent responses to items measuring detection, discrimination, and understanding of speech and environmental sounds in everyday settings.

At the same time, the present curves are grounded in caregiver reports from a Japanese sample and therefore reflect culturally and linguistically specific patterns in how listening behaviors are expressed and observed. These Japanese reference curves provide an essential baseline against which the development of children who are deaf or hard of hearing in Japan can be interpreted, rather than relying on norms derived from English-speaking populations.

Beyond their internal coherence, the Japanese curves also showed strong concordance with the English-language norms when we directly overlaid our 10th, 50th, and 90th percentile trajectories on the logistic functions reported by Cowan et al. [4]. In particular, the Japanese 50th and 90th percentile curves closely tracked the corresponding Cowan functions across the age range, with only minor differences in the timing and height of the plateau. The 10th percentile curves showed somewhat greater divergence at certain ages but retained a similar overall growth pattern. This cross-linguistic similarity of the distribution provides additional support for the construct validity of the FLI-P(J) and suggests that the underlying developmental progression of functional listening is broadly comparable in Japanese- and English-speaking children.

### 4.2. Feasibility of Survey-Based Administration and Robustness of Cleaning

A second aim of the study was to evaluate the feasibility of administering the FLI-P(J) in an online, survey-based format. Caregivers completed all 64 items using the response format (“Mostly” vs. “Rarely”), and total scores were reconstructed post hoc using a rule that mirrored the standard stop protocol. Despite this deviation from the clinical interview format, the resulting age-score relationship showed a consistent upward trend across the full age range, suggesting that caregivers were able to apply the response options consistently and that total scores behaved as expected.

Data cleaning combined clinical screening, removal of implausible zero scores, and IQR-based outlier detection within six-month age bands. A comparison of two alternative outlier-handling strategies—5th-percentile trimming plus IQR for upper outliers versus symmetric IQR trimming at both ends—showed that the trajectories were nearly identical, differing only at the extremes of the age range. This convergence indicates that the observed developmental pattern is not an artefact of a particular cleaning rule and that the final curves are robust to reasonable analytic choices.

### 4.3. Discrimination of Clinically Flagged Cases

Although children with reported medical or developmental concerns were excluded from the normative dataset, their FLI-P(J) scores were later reintroduced and plotted against the Japanese reference curves. This provided a preliminary check on whether the instrument behaves in a clinically meaningful way.

As detailed in Results and shown in Figure 4, the majority of children with identified hearing or language concerns scored at or below the 5th percentile, consistent with prior work showing that FLI-P scores are sensitive to the effects of hearing impairment and to the benefits of early intervention [3,4]. However, a subset of each group scored within or above the typical range, and one child with reported language delay scored well above the 95th percentile. This suggests that FLI-P(J) has the potential to differentiate many children with hearing or language difficulties from their typically developing peers, while also highlighting heterogeneity within clinically identified groups that cannot be fully explained with the available data. Given the small number of children with reported hearing concerns (n = 9) and the absence of independent audiological or diagnostic confirmation for either clinically flagged group, these overlays should be interpreted as preliminary evidence of the FLI-P(J)’s potential clinical applicability rather than formal validation of its diagnostic or screening properties, which will require study in an independently confirmed clinical sample.

### 4.4. Clinical and Research Implications

Taken together, the normative curves and clinical overlays provide initial evidence that FLI-P(J) is both feasible and informative as a tool for monitoring listening development in Japanese-speaking children. This kind of ongoing, quantifiable progress monitoring complements comparative research on early intervention approaches themselves [14], together supporting more individualized decision-making for children with hearing loss. For clinicians working in early intervention, cochlear implant, or hearing-aid programs, the Japanese percentile curves can serve as a reference when interpreting an individual child’s FLI-P(J) score: scores substantially below the age-appropriate percentiles may prompt closer monitoring, more intensive intervention, device management or further assessment, whereas scores within or above the typical range may provide reassurance that a child’s functional listening is on track relative to typically hearing Japanese peers.

For operational guidance, we consider the space between the 16th and 84th percentiles to represent the typical range for a given age. Scores falling below the 16th percentile may warrant additional attention and investigation; however, we emphasize that this threshold is provisional and has not yet been formally validated against independently confirmed clinical outcomes. For children whose scores fall within the typical range but toward its lower end (e.g., between the 16th and 50th percentile), we do not consider this pattern alone to indicate a clinical concern, given the normal variability inherent in caregiver report; such scores may nonetheless support a recommendation for continued monitoring rather than definitive reassurance, particularly when considered alongside other developmental indicators.

For researchers, the present baseline allows FLI-P(J) data from children with hearing impairment to be expressed in terms of age-referenced percentile ranks and, where age-specific means and standard deviations are available, as z-scores. This standardization facilitates comparison across studies and across languages. The rich set of demographic and home-environment variables collected alongside the FLI-P(J) also opens the door to future analyses examining how factors such as parental education, reading practices, and early intervention participation relate to functional listening trajectories.

### 4.5. Strengths and Limitations

Important strengths of this study include the large sample size spanning the full early childhood age range, the systematic translation and adaptation process, and the explicit comparison of alternative outlier-handling strategies. The use of an online panel enabled efficient recruitment and ensured that each month of age was represented by multiple respondents, supporting stable estimation of percentile curves.

Several limitations should also be acknowledged. First, because the normative sample was drawn from an incentivized, opt-in commercial caregiver panel, and eligibility (typical hearing, absence of developmental concerns) relied entirely on caregiver self-report without independent clinical verification, the sample is subject to potential selection bias (caregivers who join and remain active on paid panels may differ from the general population), reporting bias (caregivers may under- or over-report developmental concerns), and misclassification bias (some children screened as “typically hearing” may have undetected impairment, and vice versa). Second, all data were based on caregiver report; audiological status and developmental diagnoses were not independently verified, and we did not assess test–retest reliability or inter-rater agreement in this study. This is a limitation shared by many caregiver-completed developmental screening tools. A recent review found that psychometric properties such as reliability are inconsistently established across the field [15], underscores the need for formal reliability and validity testing of the FLI-P(J) in future work. Third, because all caregivers completed every item rather than stopping after six consecutive “Rarely” responses, the online administration format primarily differs from standard clinician administration in respondent burden rather than in the resulting score. It was not uncommon for caregivers to report a “Mostly” response to at least one item following a run of six consecutive “Rarely” responses (405 of 2512 children in the final sample, 16.1%); consistent with the standard FLI-P stop rule, these later “Mostly” responses were not counted toward the total score, since a clinician administering the assessment face-to-face would never have reached those items. The reconstructed score is therefore expected to closely approximate what would have been obtained under standard clinical administration, with the primary practical difference being the additional time required for caregivers to complete items beyond the point at which testing would typically have been discontinued. It should also be noted that a clinician experienced in administering the FLI-P may apply the “Mostly”/“Rarely” response criteria more consistently than a caregiver encountering the instrument for the first time in self-administered questionnaire form, introducing a further source of measurement variability that is not addressed by the scoring reconstruction described above. Finally, the present analyses were cross-sectional and focused on children with typical hearing; we have not yet modelled longitudinal change within individuals or formally evaluated the instrument’s sensitivity to intervention effects in Japanese children who are deaf or hard of hearing. In addition, the sequential exclusion criteria (medical screening, zero-score removal, and IQR trimming) were applied subjectively, even if based on standard practice. The zero-score exclusion in particular may have removed valid responses from children with genuine developmental delays. The present study provides preliminary normative curves and establishes feasibility, but it does not constitute a full psychometric validation.

## 5. Conclusions

Future work will extend this foundation in several directions. Item-level psychometric properties (e.g., item-response models, reliability estimates) and the sensitivity of these curves to intervention effects remain to be examined. First, FLI-P(J) should be administered longitudinally to cohorts of Japanese children with hearing impairment enrolled in early intervention and cochlear implant programs, allowing evaluation of growth trajectories relative to the normative curves and comparison with outcomes reported in English-speaking settings. Second, psychometric analyses (e.g., item-response modelling, reliability estimates) are needed to examine the internal structure of the Japanese version and to confirm that items function similarly across age and, where relevant, across subgroups. Third, the rich contextual data collected in the present survey can be used to explore how demographic and home-environment factors relate to functional listening, potentially identifying modifiable aspects of the child’s environment that support listening development. Finally, building on the existing digital FLI-P platform already in use in clinical programs, a Japanese-language module incorporating the FLI-P(J) items and norms could facilitate routine monitoring, automated score calculation relative to Japanese reference data, and user-friendly visual feedback for clinicians and parents in Japan to understand a child’s listening development. As access to digital FLI-P tools is expanded beyond clinicians to parents and caregivers, integrating FLI-P(J) into these systems would also enable more direct, family-facing monitoring of functional listening development. Future validation should include three pillars: (1) recruiting children with confirmed clinical diagnoses to verify curve sensitivity; (2) prospective longitudinal tracking of children scoring below the 16th percentile; and (3) examining the predictive validity of the FLI-P(J) for later Japanese language outcomes.

In summary, this study provides preliminary normative reference curves for the Japanese version of the Functional Listening Index—Paediatric and demonstrates the feasibility of administering FLI-P(J) in an online caregiver-survey format. The resulting baseline offers a crucial starting point for applying FLI-P(J) in Japanese clinical and research contexts and for evaluating the listening development of children with hearing impairment relative to their typically hearing peers.

## Figures and Tables

**Table 1:** Number of caregiver responses per six-month age band before and after data cleaning (see Section 2.4 for exclusion criteria).

Age Bin	Total Participants	After Cleaning	Percentage
2–7	219	195	89.04
8–13	243	214	88.07
14–19	255	224	87.84
20–25	251	227	90.44
26–31	251	218	86.85
32–37	249	221	88.76
38–43	249	203	81.53
44–49	252	217	86.11
50–55	251	198	78.88
56–61	252	198	78.57
62–67	251	200	79.68
68–73	253	197	77.87
	2976	2512	

## Data Availability

The complete Japanese translation of the survey instrument (FLI-P(J)) used in this study, along with the resultant dataset, is available online in the accompanying Mendeley Data repository [https://doi.org/10.17632/jcvw93k8kj.1].

## Abbreviations

The following abbreviations are used in this manuscript:

FLI-P	Functional Listening Index—Paediatric
FLI-P(J)	Functional Listening Index—Paediatric (Japan)

## References

[1] (2019). Functional Listening Index—Paediatric^®^—The Shepherd Centre. https://shepherdcentre.org.au/how-we-help/programs-and-services/download-functional-listening-index-paediatric/.

[2] (2020). FLI-P^®^—Hearhub. https://hearhub.org/hearhub-features/fli-p/.

[3] Davis A., Harrison E., Cowan R. (2022). The Feasibility of the Functional Listening Index—Paediatric (FLI-P^®^) for Young Children with Hearing Loss. J. Clin. Med..

[4] Cowan R.S.C., Davis A., Watkins P., Neal K., Brookman R., Seeto M., Oliver J. (2024). Tracking Listening Skill Development in Infants and Children with Hearing Loss: A Normative Dataset for the Functional Listening Index—Paediatric (FLI-P^®^). Children.

[5] Haris S.M.B. (2025). The Functional Listening Index For Pediatric (FLI-P): Translation and Adaptation into the Malay Language and Psychometric Properties Evaluation. Kuantan Pahang Kulliyyah Allied Health Sci. Int. Islam. Univ. Malays.

[6] Oshima-Takane Y., Kobayashi T., Chan E. (2025). Learning novel transitive verbs in causative action events: A cross-linguistic comparison between English- and Japanese-speaking infants. J. Exp. Child. Psychol..

[7] Childers J.B., Imai M., Ohba M., Perry F., Marsh L. (2025). Examining children’s verb learning in the United States and Japan: Do comparisons help?. J. Exp. Child. Psychol..

[8] Imai M., Li L., Haryu E., Okada H., Hirsh-Pasek K., Golinkoff R.M., Shigematsu J. (2008). Novel Noun and Verb Learning in Chinese-, English-, and Japanese-Speaking Children. Child Dev..

[9] Doi N., Fukunaga I., Kobayashi T., Hirose K., Hyodo M., Teshima M. (2025). Impact of the Improved Publicly-funded Newborn Hearing Screening Program. JMA J..

[10] Beaton D.E., Bombardier C., Guillemin F., Ferraz M.B. (2000). Guidelines for the Process of Cross-Cultural Adaptation of Self-Report Measures. Spine.

[11] Dash C.S.K., Behera A.K., Dehuri S., Ghosh A. (2023). An outliers detection and elimination framework in classification task of data mining. Decis. Anal. J..

[12] The jamovi Project (2025). jamovi.

[13] Flegal K.M. (2013). Construction of LMS Parameters for the Centers for Disease Control and Prevention 2000 Growth Charts. National Health Statistics Reports.

[14] Casoojee A., Khoza-Shangase K., Kanji A. (2025). Communication Outcomes of Children with Hearing Loss: A Comparison of Two Early Intervention Approaches. Audiol. Res..

[15] Kurbatfinski S., Komanchuk J., Dosani A., Letourneau N. (2024). Validity, Reliability, Accessibility, and Applicability of Young Children’s Developmental Screening and Assessment Tools across Different Demographics: A Realist Review. Children.

